# Comprehensive Plasma Metabolomic Analyses of Atherosclerotic Progression Reveal Alterations in Glycerophospholipid and Sphingolipid Metabolism in Apolipoprotein E-deficient Mice

**DOI:** 10.1038/srep35037

**Published:** 2016-10-10

**Authors:** Vi T. Dang, Aric Huang, Lexy H. Zhong, Yuanyuan Shi, Geoff H. Werstuck

**Affiliations:** 1 Thrombosis and Atherosclerosis Research Institute, Hamilton, Ontario, Canada; 2 Department of Chemistry and Chemical Biology, McMaster University, Hamilton, Ontario, Canada; 3 Department of Medicine, McMaster University, Hamilton, Ontario, Canada

## Abstract

Atherosclerosis is the major underlying cause of most cardiovascular diseases. Despite recent advances, the molecular mechanisms underlying the pathophysiology of atherogenesis are not clear. In this study, comprehensive plasma metabolomics were used to investigate early-stage atherosclerotic development and progression in chow-fed apolipoprotein E-deficient mice at 5, 10 and 15 weeks of age. Comprehensive plasma metabolomic profiles, based on 4365 detected metabolite features, differentiate atherosclerosis-prone from atherosclerosis-resistant models. Metabolites in the sphingomyelin pathway were significantly altered prior to detectable lesion formation and at all subsequent time-points. The cytidine diphosphate-diacylglycerol pathway was up-regulated during stage I of atherosclerosis, while metabolites in the phosphatidylethanolamine and glycosphingolipid pathways were augmented in mice with stage II lesions. These pathways, involving glycerophospholipid and sphingolipid metabolism, were also significantly affected during the course of atherosclerotic progression. Our findings suggest that distinct plasma metabolomic profiles can differentiate the different stages of atherosclerotic progression. This study reveals that alteration of specific, previously unreported pathways of glycerophospholipid and sphingolipid metabolism are associated with atherosclerosis. The clear difference in the level of several metabolites supports the use of plasma lipid profiling as a diagnostic tool of atherogenesis.

Cardiovascular disease (CVD) is the leading cause of morbidity and mortality worldwide^1^,^2^. Risk factors of CVD include obesity, dyslipidemia, stress, smoking, hypertension and diabetes mellitus^3^. The major underlying cause of most CVD is atherosclerosis, which is a multifactorial and progressive disease of the medium-large arteries^4^. Atherosclerosis is characterized by the accumulation of lipids and inflammatory factors in the arterial walls. Despite recent advances, the molecular mechanism(s) underlying the pathogenesis and progression of atherosclerosis are still not completely understood^5^,^6^,^7^. This has complicated the diagnosis, treatment and prevention of atherosclerosis-related diseases.

Metabolomics, which is the study of small molecules, offers a novel approach to investigate disease mechanisms and identify disease biomarkers, as it is capable of providing a global snapshot of the dynamic intracellular changes associated with a particular physiological or pathological state^8^,^9^. Moreover, the levels of metabolites can more accurately reflect the functional status of an organism compared to the other ‘omics’ studies, such as genomics or proteomics, because metabolic fluxes are regulated not only by gene expression, but also by environmental stresses^10^. Therefore, the status of specific metabolite levels is a critical indicator of human health or disease.

In this study, comprehensive metabolomic techniques were used to investigate atherosclerosis in chow-fed apolipoprotein E-deficient (ApoE^−/−^) mice at 5, 10 and 15 weeks of age in order to discover novel metabolic pathways and metabolisms associated with early stages of atherosclerotic development and progression. Comprehensive metabolomics involves the global analysis of the metabolome^10^,^11^. This type of approach is capable of revealing novel and unanticipated molecular perturbations as it represents an unbiased examination of the association between the levels of metabolites and their interconnectivity in multiple metabolic pathways, with relation to a phenotype or genotype.

The ApoE^−/−^ mouse model was chosen to investigate atherosclerosis because it is a well-established model in which all recognized stages of atherogenesis can be observed^12^,^13^. In addition, the complexity and morphological features of atherosclerotic lesions that develop in this mouse model are very similar to those in humans^14^,^15^. Atherosclerosis in ApoE^−/−^ mice is driven by impaired clearance of cholesterol-enriched lipoproteins, which results in elevated levels of plasma cholesterol and atherogenic remnants. In this study, mice were fed a standard chow diet to avoid other metabolic complications associated with high fat diet including weight gain, insulin resistance and non-alcoholic fatty liver disease. We chose to investigate early stages of atherogenesis to address current limitations in our understanding of how the pathogenesis of atherosclerosis initiates, as well as the lack of diagnostic tools for early-stage detection of this disease.

## Results

### Atherosclerosis progresses with aging in ApoE^−/−^ mice

No significant differences were observed in body weight, fasting blood glucose or plasma triglycerides levels between atherosclerosis-prone homozygous ApoE^−/−^ mice and age-matched atherosclerosis-resistant heterozygous ApoE^+/−^ mice (Supplementary Table S1). Plasma total cholesterol, as well as very low-density, intermediate-density and low-density lipoprotein cholesterol were significantly elevated in ApoE^−/−^ mice, relative to ApoE^+/−^ mice, at all ages (Supplementary Fig. S1). These atherogenic lipoproteins were undetectable in the control ApoE^+/−^ mice. ApoE^−/−^ mice also showed a moderate decrease in high density lipoprotein levels, compared to ApoE^+/−^ mice at all ages.

**Figure 1:**
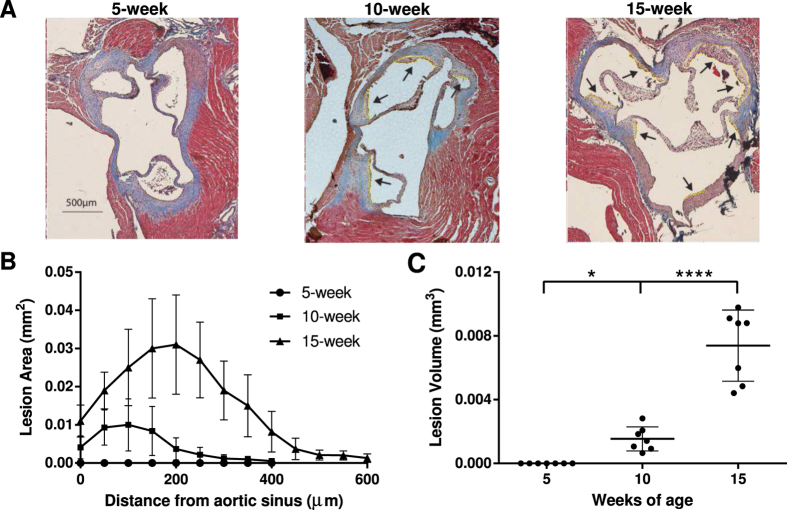
Atherosclerotic progression in ApoE^−/−^ mice. (**A**) Representative images of aortic cross-sections of 5, 10 and 15-week-old mice stained with Masson’s trichrome. Atherosclerotic lesions are indicated by arrows and outlined in yellow. Quantification of atherosclerotic lesion area (**B**) and volume (**C**) of 5, 10 and 15-week-old ApoE^−/−^ mice. n = 7/group, *p < 0.05, ****p < 0.0001. Data are presented as the mean ± SD.

**Figure 2:**
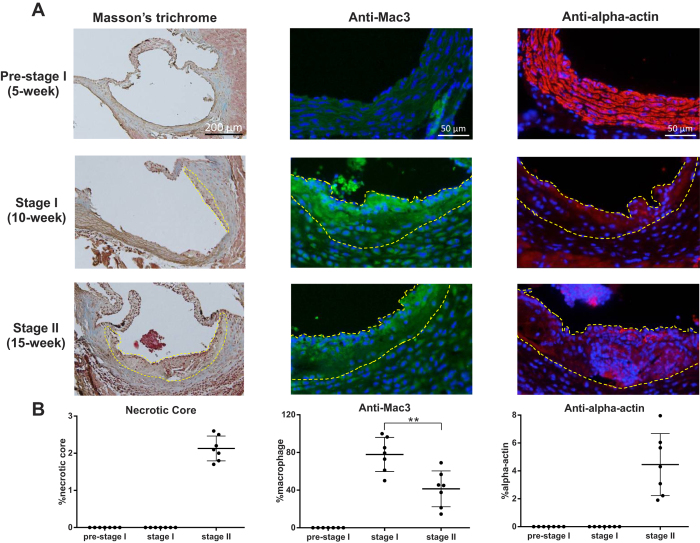
Characterization of atherosclerotic progression in ApoE^−/−^ mice. (**A**) Representative images of aortic cross-sections of ApoE^−/−^ mice at 5, 10 and 15 weeks of age stained with the indicated chemicals or antibodies. Atherosclerotic plaques are outlined by the dashed yellow line. (**B**) Quantification of necrotic core, macrophage and intimal smooth muscle cell content in atherosclerotic lesions found in ApoE^−/−^ mice at pre-stage I, stage I and stage II of atherosclerosis. Percentages were calculated by dividing positively stained areas by total lesion area. n = 7/group, **p < 0.01. Data are presented as the mean ± SD. Blue: DAPI, green: macrophage, red: smooth muscle cell.

Atherosclerotic lesions were not detected in any of the 5-week-old ApoE^−/−^ mice (Fig. 1) or at any age in ApoE^+/−^ mice (Supplementary Fig. S2). Fatty streaks were observed in ApoE^−/−^ mice at 10 weeks of age while more advanced lesions, with areas of necrosis and crystallized cholesterol, were detected in 15-week-old ApoE^−/−^ mice (Fig. 2). Atherosclerotic lesions in the aortic sinus of 15-week-old ApoE^−/−^ mice were approximately 4.2 fold larger (*p*-value < 0.0001) than those in 10-week-old ApoE^−/−^ mice (Fig. 1).

Lesional macrophage/foam cell content, adjusted to lesion area, was significantly elevated in ApoE^−/−^ mice at 10 weeks, relative to 15 weeks of age (Fig. 2). Intimal smooth muscle cells, collagen and apoptotic cells, which are indicative of more complex and advanced lesions, were detected only in 15-week-old ApoE^−/−^ mice (Fig. 2 and Supplementary Fig. S3). Based upon these morphological features, atherosclerotic lesions detected in 10- and 15-week-old ApoE^−/−^ mice were classified as stage I (early) and stage II (intermediate) lesions, respectively^12^,^14^.

### Comprehensive plasma metabolomic profiles differentiate atherosclerosis-prone and atherosclerosis-resistant models

Mouse plasmas were extracted and analyzed by liquid chromatography coupled to a mass spectrometer. We detected 2435 and 1930 metabolite features (n = 4365 total) in negative and positive ionization modes, respectively. The principle component analysis score plot showed a tight clustering of quality control samples (Supplementary Fig. S4), indicating good instrumental reproducibility throughout the period of analysis.

**Figure 3:**
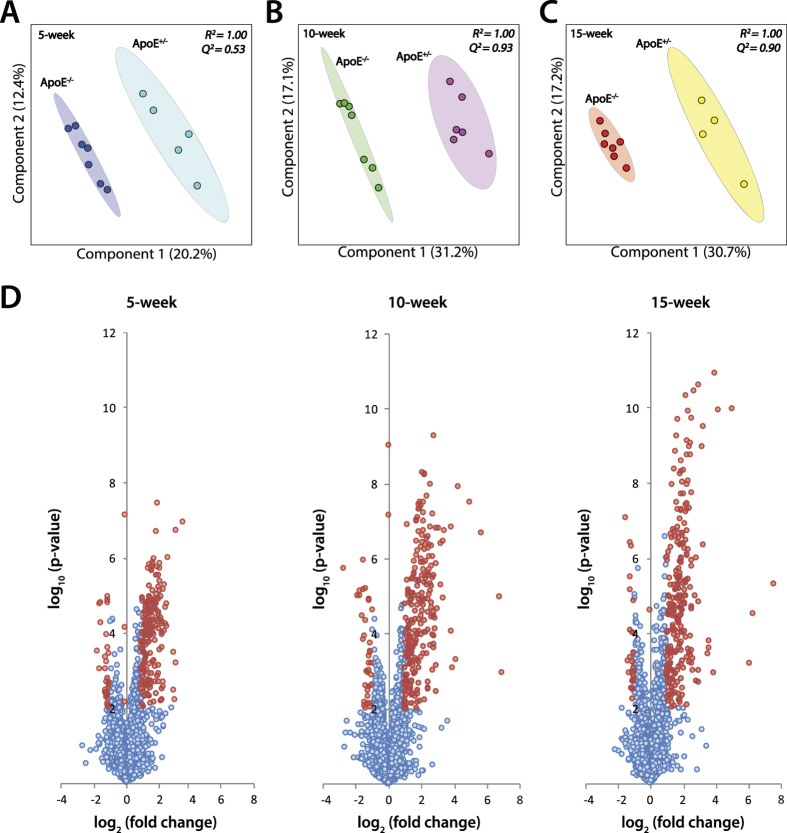
Comprehensive plasma metabolomic profiles differentiate atherosclerosis-prone and atherosclerosis-resistant models. Direct comparison of the PLS-DA score plots of 4365 total metabolite features detected in plasma of (**A**) 5-week, (**B**) 10-week and (**C**) 15-week ApoE^−/−^ mice versus age-matched ApoE^+/−^mice. Each dot represents the plasma metabolomic profile of a single mouse. R^2^ and Q^2^ indicate prediction accuracy and model robustness, respectively. (**D**) Volcano plots indicating the number of metabolite features that were significantly altered between ApoE^−/−^ and ApoE^+/−^ mice at 5, 10 and 15-week time points. Each plot encompasses 4365 metabolite features. Red dots indicate metabolite features with *p* < 0.01 and fold change >±2. n = 7 per ApoE^−/−^ group and n = 4–6 per ApoE^+/−^ group.

Multivariate analyses were used to correlate the metabolomic variables with genotypic and phenotypic patterns. The comprehensive plasma metabolomic profiles of ApoE^−/−^ mice at pre-stage I, stage I and stage II of atherosclerosis were clearly separated from those of age-matched ApoE^+/−^ mice in the partial least squares discriminant analysis (PLS-DA) plot constructed based on the 4365 total metabolite features (Fig. 3A–C). At five weeks of age, prior to any detectable phenotypic differences, the metabolomic profile of ApoE^−/−^ mice was already distinct from that of ApoE^+/−^ mice with moderate prediction accuracy (Q^2^ of 0.53). The prediction accuracy increases significantly at 10 and 15-week time points with Q^2^ value of 0.93 and 0.90 respectively, relative to the 5-week time point. A Q^2^ value greater than 0.9 indicates a highly robust multivariate model. Similarly, the separation component between the metabolomic profiles of ApoE^−/−^ and ApoE^+/−^ mice (component 1 in the PLS-DA score plot) also increases substantially from 5 to 10 and 15-week time points (20.2% to 31.2% and 30.7%, respectively).

Volcano plots were used to visualize the number of metabolite features that were significantly altered between ApoE^−/−^ and age-matched ApoE^+/−^ mice (Fig. 3d). Over 200 metabolite features were found to be significantly altered (*p*-value < 0.01 and fold change >2) between the two mouse models at each time point. At a more extreme significance cut-offs (*p*-value < 0.0001 and fold change >5), we detected 12, 29 and 51 altered metabolite features at the 5-, 10- and 15-week time point, respectively. This indicates that the number of newly emerging significant features, i.e., not observed in the earlier time-point, also increases substantially from pre-stage I to stage I and stage II of atherosclerosis. These findings are consistent with the clinical and physiological observation of the progressive phenotypic differences in atherosclerotic progression.

### Identification of significantly altered plasma metabolites between the atherosclerosis-prone and age-matched atherosclerosis-resistant models

**Figure 4:**
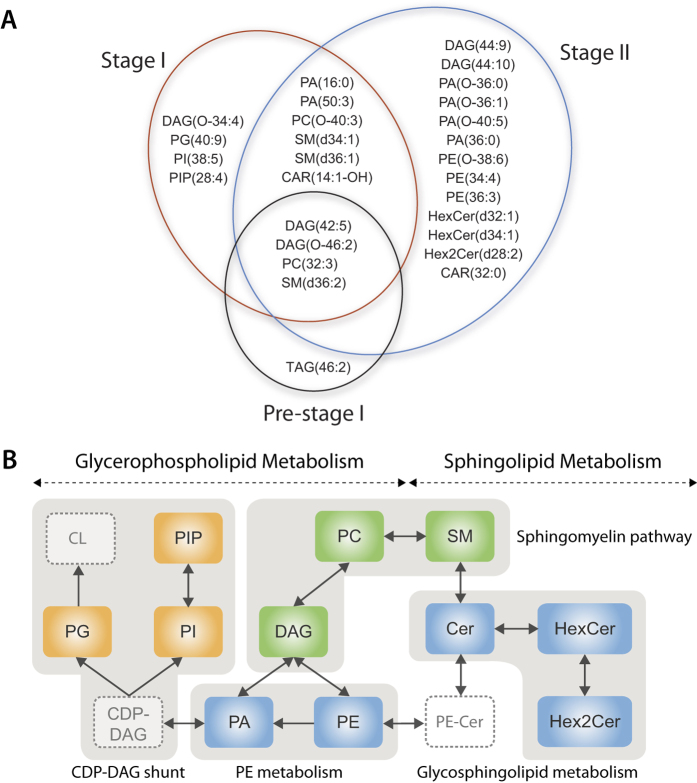
Identification of significantly altered metabolites between the atherosclerosis-prone and age-matched atherosclerosis-resistant models. (**A**) Venn diagram showing extremely altered metabolites (*p* < 0.0001 and fold change >±5) that were uniquely altered and shared between pre-stage I, stage I and stage II of atherosclerosis. (**B**) Extremely altered metabolites were mapped into metabolic pathways. Metabolites in green were altered at all three time points, whereas those in orange and blue were altered at 10 and 15-week time point, respectively. The dashed box indicates metabolites that were not detected in the analysis. CAR: acylcaritine, CDP: cytidine diphosphate, Cer: ceramide, CL: cardiolipin, DAG: diacylglycerol, HexCer: hexosyl-ceramide, PA: phosphatidic acid, PC: phosphatidylcholine, PE: phosphatidylethanolamine, PG: phosphatidylglycerol, PI: phosphatidylinositol, PIP: PI-phosphate, SM: sphingomyelin, TAG: triacylglycerol.

A Venn diagram, summarizing the identification of extremely different metabolites (*p*-value < 0.0001 and fold change >5) between ApoE^−/−^ and age-matched ApoE^+/−^ mice, is shown in Fig. 4A and Supplementary Table S2. The concentrations of several species of diacylglycerol, phosphatidylcholine and sphingomyelin were altered at all three time points in ApoE^−/−^ mice, compared to age-matched ApoE^+/−^ mice. These metabolites are involved in the sphingomyelin biosynthetic pathway (Fig. 4b).

The levels of phosphatidylglycerol, phosphatidylinositol and phosphatidylinositol-phosphate were uniquely elevated at 10-week point in ApoE^−/−^ mice, relative to ApoE^+/−^ mice. This suggests an up-regulation in the cytidine diphosphate-diacylglycerol pathway (glycerophospholipid metabolism) during the early stage of atherosclerotic progression (Fig. 4B). At the 15-week time point, we observed an alteration in the concentration of several species of hexosyl-ceramide, dihexosyl-ceramide and phosphatidylethanolamine between the two mouse models. These results suggest that glycosphingolipid (sphingolipid metabolism) and phosphatidylethanolamine pathways (glycerophospholipid metabolism) are altered during the intermediate stage of atherosclerotic progression (Fig. 4B).

Overall, the majority of the metabolites showing the greatest variation between the atherosclerosis-prone and atherosclerosis-resistant models are involved in glycerophospholipid and sphingolipid metabolism. This suggests that these pathways play important roles in the pathogenesis and progression of early stage atherosclerosis. Approximately half of the extremely altered metabolite features remain unidentified.

### Select plasma metabolomic profiles distinguish different stages of atherosclerotic progression

In order to further investigate the metabolic changes associated with atherosclerotic progression, we examined the metabolite variations over the three stages of atherosclerosis observed in ApoE^−/−^ mice at 5, 10 and 15 weeks of age.

**Figure 5:**
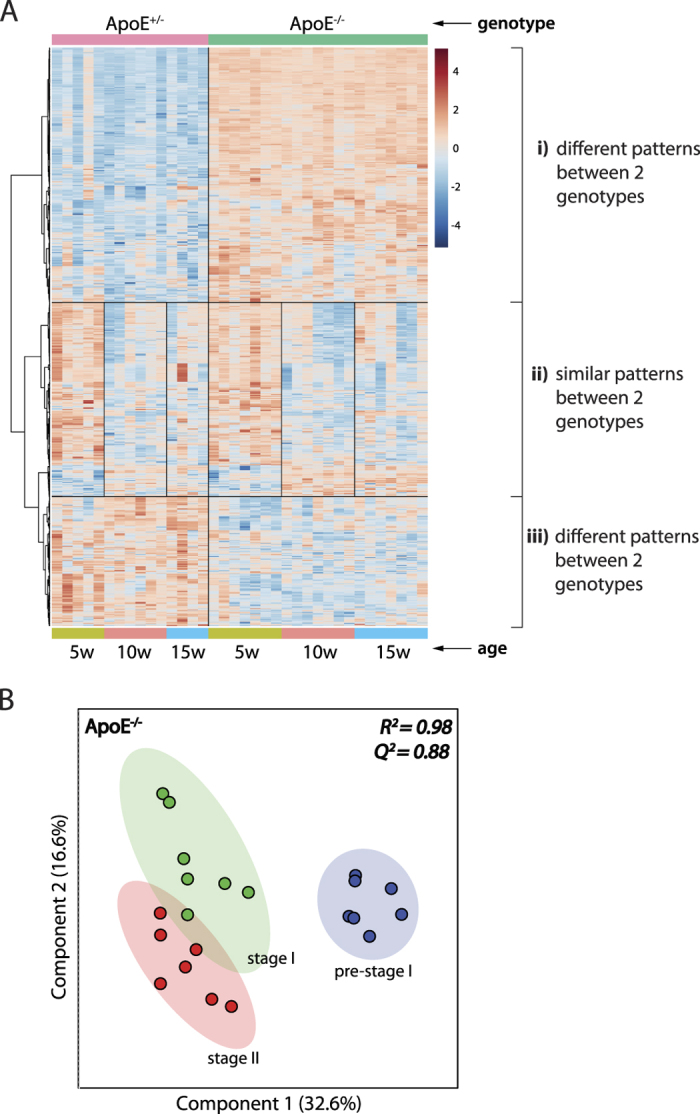
Select plasma metabolomic profiles distinguish different stages of atherosclerotic progression in ApoE^−/−^ mouse model. (**A**) Heat map indicating the relative levels of the top 2000 metabolite features ranked by between-subject two-way ANOVA *p*-value. The overall data is depicted in terms of genotype and age. Metabolites that exhibit similar (ii) or different patterns (i, iii) between the two genotypes are indicated. Each line represents a single metabolite, coloured by its abundance (red: high abundance and blue: low abundance). (**B**) PLS-DA score plot of 137 significantly altered metabolites (*p* < 0.01) detected in plasma of ApoE^−/−^ mice at pre-stage I, stage I and stage II of atherosclerosis. Each dot represents the plasma metabolomic profile of a single mouse. R^2^ and Q^2^ indicate prediction accuracy and model robustness, respectively. n = 7/group.

A heat map of the top 2000 metabolite features ranked by *p*-value (two-way ANOVA) is presented in Fig. 5A. This analysis depicts the overall data structure in terms of two factors: genotype and age. A specific subset of metabolite features clearly differentiates the ApoE^−/−^ and ApoE^+/−^ genotypes, independent of age (Fig. 5A-i, iii). A separate subset of features displays similar patterns throughout the three time points, between the two genotypes (Fig. 5A-ii). The features that are similarly altered in both mouse models are most likely associated with age, rather than the pathophysiology of the disease. These age-associated metabolite features were removed to ensure that all detectable metabolic changes are most closely associated to atherosclerotic progression.

A total of 137 metabolites were identified to be significantly altered (ANOVA *p*-value < 0.01) between the three atherosclerotic stages in ApoE^−/−^ mice, after correcting for age-associated features (Supplementary Table S3). Select plasma metabolomic profiles, constructed based on these significantly altered metabolites, differentiate pre-stage I, stage I and stage II of atherosclerosis in ApoE^−/−^ mice (Fig. 5B) with very good prediction accuracy (Q^2^ value of 0.88). The observed separation trend was consistent with the progression of atherosclerosis in ApoE^−/−^ mice from pre-stage I to stage I and stage II of atherosclerosis.

### Pathway analysis of atherosclerotic progression

**Figure 6:**
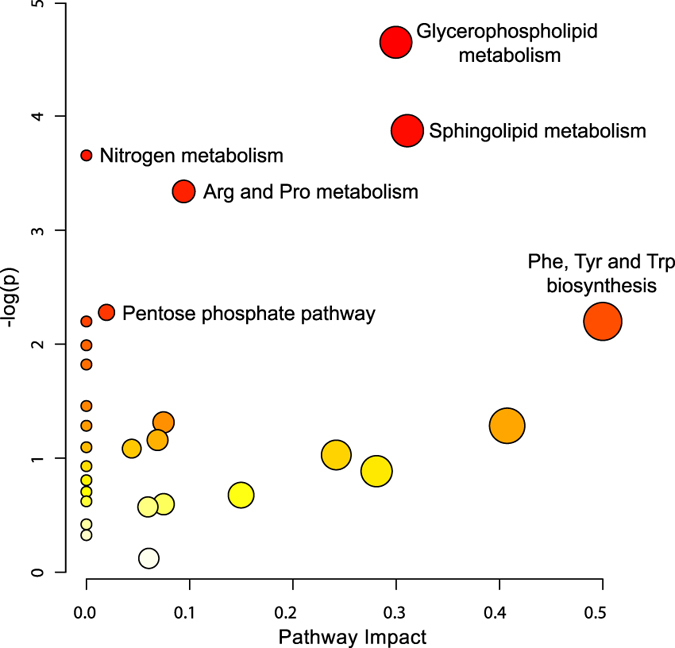
Pathway analysis of atherosclerotic progression. Pathway analysis was performed on the 137 significantly altered metabolites (*p* < 0.01) between the three stages of atherosclerosis observed in ApoE^−/−^ mice. All matched pathways are plotted according to *p*-value from pathway enrichment analysis and pathway impact score from pathway topology analysis. Colour gradient and circle size indicate the significance of the pathway ranked by *p*-value (yellow: higher *p*-values and red: lower *p*-values) and pathway impact score (the larger the circle the higher the impact score), respectively. Significantly affected pathways with low *p*-value and high pathway impact score are identified by name.

Pathway analysis was performed on the 137 significantly altered plasma metabolites between the three atherosclerotic stages observed in ApoE^−/−^ mice. The results from integrating enrichment and pathway topology analyses^16^ were used to map out the identified metabolites into specific metabolic pathways. On this basis, 29 pathways were found to be potentially affected during the progression of atherosclerosis (Fig. 6, Supplementary Table S4).

**Figure 7:**
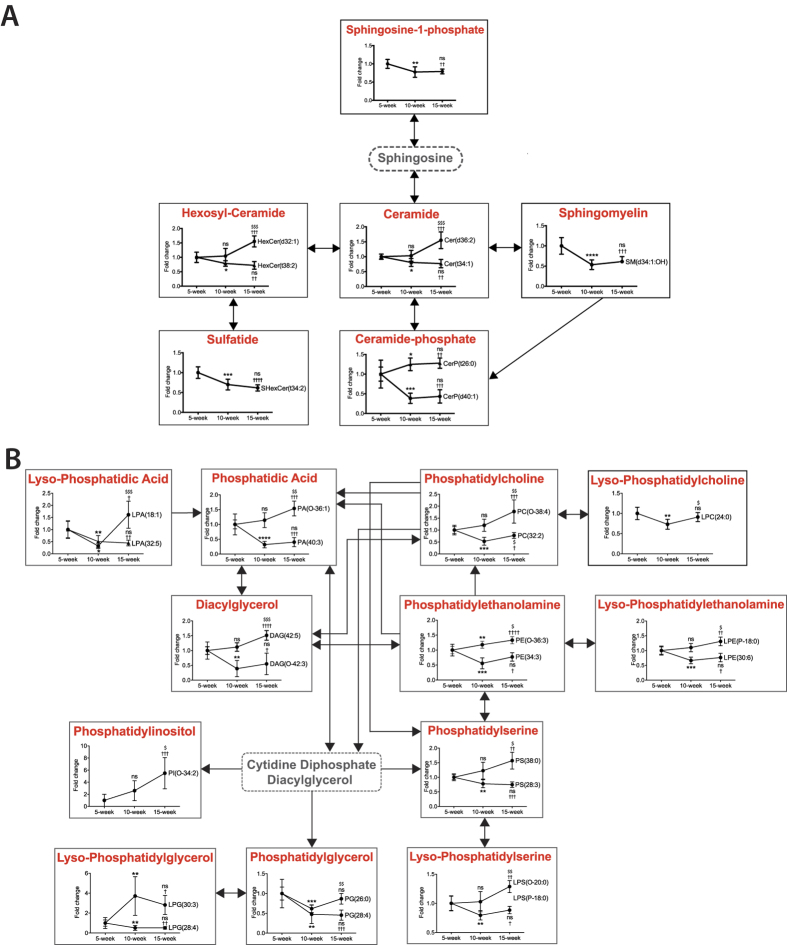
Sphingolipid and glycerophospholipid metabolism. Metabolites from (**A**) sphingolipid and (**B**) glycerophospholipid metabolism were mapped into respective metabolic pathways. All metabolites presented were significantly altered (ANOVA *p* < 0.01) between the three atherosclerotic stages detected in ApoE^−/−^ mice. Each lipid species is showed in one box. In the case of within-species metabolites display different alteration trends between the three stages of atherosclerosis; two metabolites (randomly selected) are shown. Metabolites encircled by a dashed line (sphingosine and cytidine diphosphate diacylglycerol) were not detected. Data are presented as fold change relative to the level of the indicated metabolite detected in plasma of 5-week-old ApoE^−/−^ mice. n = 7/group; *5-week vs. 10-week, †5-week vs. 15 week, ^$^10-week vs. 15-week; 1, 2, 3 and 4 asterisks indicate *p* < 0.05, <0.01, <0.001 and <0.0001, respectively. Data are presented as the mean ± SD.

Glycerophospholipid and sphingolipid metabolism were again the most significantly affected pathways based on *p*-values and pathway impact scores. Other impacted pathways included arginine and proline metabolism (amino acid metabolism), nitrogen metabolism (energy metabolism) and pentose phosphate pathway (carbohydrate metabolism). Metabolites from sphingolipid (Fig. 7A) and glycerophospholipid (Fig. 7B) metabolism were mapped into respective metabolic pathways. Most of the key intermediate metabolites involved in these metabolisms were significantly altered between the three stages of atherosclerosis observed in ApoE^−/−^ mice.

## Discussion

The objective of this study was to identify alterations in specific plasma-borne metabolites that are associated with the pathogenesis of atherosclerosis. Since atherosclerosis is a progressive disease, age-associated confounding features must be considered in the investigation of disease progression. As such, age-matched atherosclerosis-resistant ApoE^+/−^ littermates were used as controls to account for age-related metabolic changes. The detectable molecular alterations can be attributed to the increase in plasma cholesterol and associated with the pathophysiology of atherosclerosis. This study was performed with female mice because they have faster and more pronounced early atherosclerotic progression, compared to age-matched male ApoE^−/−^ mice^17^.

The development and progression of atherosclerosis observed in this study was consistent with previous results in chow-fed ApoE^−/−^ mice^12^,^14^. Comprehensive plasma metabolomic profiles differentiated atherosclerosis-prone and atherosclerosis-resistant models. The degree of separation in the metabolomic profiles, as well as the number of extremely altered metabolites between the two mouse models increased substantially from 5 to 10 and 15-week time points, which mirrors the progressive differences in the phenotypes between ApoE^−/−^ and ApoE^+/−^ mice over time. Identification of significantly altered metabolites between the atherosclerosis-prone and age-matched atherosclerosis-resistant models revealed changes in pathways involving glycerophospholipid and sphingolipid metabolism. These lipids are not only structural components of biological membranes, but also act as signaling molecules and bioactive mediators in important atherosclerosis-related cellular processes, including apoptosis, angiogenesis, inflammation and proliferation^18^,^19^,^20^,^21^,^22^. Disturbances in their metabolism have been previously observed in atherosclerosis-related diseases^23^,^24^,^25^; however, to the best of our knowledge, we are the first group to identify the specific pathways within these metabolic systems that are associated with the specific stages of atherosclerotic progression.

Metabolites in the sphingomyelin biosynthetic pathway are significantly altered prior to detectable lesion formation and at all subsequent time points. This pathway is an important link between glycerophospholipid and sphingolipid metabolism. Phosphatidylcholine and sphingomyelin are the two most abundant phospholipids in mammalian plasma^26^,^27^. These phospholipids also appear in all major lipoproteins and are abundant in atherosclerotic lesions. Both plasma phosphatidylcholine and sphingomyelin have been identified as independent risk factors for coronary heart disease^28^,^29^,^30^,^31^.

Metabolites in the glycosphingolipid pathway were augmented in ApoE^−/−^ mice with intermediate lesions. Glycosphingolipids are involved in the hydrolytic pathway of ceramide metabolism. Altered levels of hexosyl-ceramide suggests a dysregulation of ceramide synthase, which is an important mediator of sphingolipid metabolism and a key regulator of endoplasmic reticulum (ER) homeostasis^32^. Indeed, increased levels of hexosyl-ceramide have been associated with the upregulation of C/EBP homologous protein (CHOP), an indicator of ER stress^33^,^34^. Previous findings from our lab and others have implicated ER stress in the development and progression of atherosclerosis^35^,^36^,^37^,^38^.

We further investigated the progression of atherosclerosis within the atherosclerosis-prone model. Atherosclerotic progression has previously been explored at the metabolomics level^39^,^40^. However, these studies did not appropriately account for age-associated confounding metabolites in disease progression. Moreover, they did not use a high-throughput, high-sensitivity and comprehensive analytical system, as in this study. This system is capable of simultaneously detecting a wide range of polar and nonpolar molecules from complex mixtures such as bio-fluids^41^.

After removing age-associated confounding metabolite features, 29 metabolic pathways associated with multiple metabolisms were found to be potentially affected during the progression of atherosclerosis within ApoE^−/−^ mice. These findings are consistent with our understanding of the multifactorial nature of atherosclerosis, which may reflect the multiple cellular and molecular pathways involved in the development and progression of this disease^4^,^6^,^7^. Glycerophospholipid and sphingolipid metabolism were again the most significantly altered pathways during the progression of atherosclerosis. We also observed a disturbance in arginine metabolism. The level of plasma arginine decreased with atherosclerotic progression. Depletion of arginine is an indicator of systemic inflammation, which is the hallmark of all stages of atherosclerosis^42^,^43^.

Since lipids are readily interconverted by complex enzymatic machineries, their specific roles and functions are structure and context specific. In this study, several metabolites from the same lipid species showed different trends of alteration between the three stages of atherosclerosis. For example, within the ceramide species, the concentration of those with 1,3-*di*hydroxy long-chain bases (‘d’ designation) increased with atherosclerotic progression, whereas those with 1,3,4-*tri*hydroxy (‘t’ designation) showed opposite trend. Thus, despite belonging to the same lipid species, metabolites with different structural configuration, degree of saturation and/or length of carbon chain may exert different biological functions.

Lipids have diverse biological roles and their metabolism is extremely complex. As such, it is possible that no single lipid species will be suitable as a disease biomarker. Profiling several lipid species may be of more value in predicting the development and progression of a disease. In this study, the clear difference in the plasma levels of several metabolites, between the atherosclerosis-prone and atherosclerosis-resistant models, supports the feasibility of this concept. Metabolites that are altered prior to detectable lesion formation have the potential to be predictive biomarkers that may be used to facilitate the detection of the disease prior to the appearance of clinical phenotypes. Alternatively, metabolites that were uniquely altered at stage I or stage II of atherosclerosis could potentially be used as prognostic biomarkers in order to aid in the identification of the specific stages of disease progression.

This study identified systemic changes of specific plasma metabolites that are associated with the progression of atherosclerosis in ApoE^−/−^ mice. These molecular alterations are mainly attributed to the increase in plasma cholesterol and atherogenic remnant lipoproteins that can be associated with the pathophysiology of atherosclerosis. As the present study was designed to identify metabolites that are associated with atherogenesis, future studies should explore the cause-effect nature of the relationship of these metabolites with atherogenesis in order to determine the roles of these pathways in the development and progression of this disease. In addition, knowledge of the origin (i.e. tissue localization) and source (i.e. free or lipoprotein-associated lipid) of the altered metabolites will require further investigation. As there are no human equivalents to the atherogenic remnant lipoproteins that accumulate in the plasma of ApoE^−/−^ mice, the findings in this study are potentially dependent on the lipoprotein profiles of this mouse model. The relevance of these changes in other models of atherosclerosis as well as in plasma samples from humans with and without atherosclerosis will be explored.

In conclusion, dysregulation of lipid metabolism has been associated with numerous pathological conditions including cardiovascular diseases^44^,^45^. Therefore, lipid metabolism has been the target for the prognosis, diagnosis and treatment of these diseases. Based on the results of this study, alterations in glycerophospholipid and sphingolipid metabolism may be a metabolic footprint of atherosclerotic progression. As such, they represent potential targets for further investigation. Our data also suggests that specific branches of these two pathways may be involved at different stages of atherosclerosis. Compared to cholesterol and glycerolipids, our knowledge of the roles of sphingolipid and glycerophospholipid in atherosclerosis is still very limited. Future research will explore the role(s) of each lipid class and individual lipids in atherogenesis, as well as their relevance in human patients with cardiovascular disease.

## Methods

### Animal models

ApoE^−/−^ mice were crossed with ApoE^+/−^ mice to produce ApoE^−/−^ and control ApoE^+/−^ littermates. Litters were genotyped and randomly assigned into age groups. Four week old female ApoE^−/−^ and ApoE^+/−^ mice (B6.129P2-ApoE^tm1Unc^) in a C57BL6 genetic background were fed a chow diet (TD92078, Harlan Teklad, WI) *ad libitum* for 1, 6 or 11 weeks and maintained on a 12-hour light/dark cycle throughout the study. Subsets of mice were sacrificed at 5, 10 and 15 weeks of age (n = 7 per ApoE^−/−^ group and n = 4–6 per ApoE^+/−^ group). Mice were fasted for 6 hours and anaesthetized with 3% isoflurane. Blood was collected by cardiac puncture and mice were euthanized by cervical dislocation. Vasculature was flushed with saline and tissues were collected and prepared for analyses. Plasma total cholesterol and triglycerides were measured using Infinity reagents (Thermo-Scientific). Plasma lipoproteins were separated by fast protein lipid chromatography and total cholesterol was measured in each fraction using the Infinity cholesterol assay. All animal procedures were approved by and performed in accordance with the McMaster University Animal Research Ethics Board and conform with the guidelines of the Canadian Council on Animal Care.

### Histochemistry and immunofluorescence analyses

Harvested hearts were fixed in formalin. Aortas, including aortic sinus, were sectioned (5 μm/section), as previously described^46^. Atherosclerotic lesions, necrotic core and lesion collagen content were visualized and quantified by staining serial sections with Masson’s Trichrome (Sigma-Aldrich). Apoptotic cell death was measured according to the manufacturer’s instructions (Promega DeadEnd^TM^ Fluorometric TUNEL System). Different serial sections were stained with primary antibodies against the macrophage marker CD107b (Mac3, BD Pharmingen) or the vascular smooth muscle cell marker α-actin (Santa Cruz). Negative controls were stained with pre-immune rat IgG (Vector) or mouse IgG (Invitrogen), instead of primary antibodies, to correct for non-specific staining. Sections were stained with the nuclear counterstain 4′,6-Diamidino-2-phenylindole dihydrochloride (DAPI, Sigma). Stained sections were imaged using a Leitz LABORLUX S microscope connected to a DP71 Olympus camera. Lesion area and positively immunostained areas were quantified using Image J (1.48v) software. Lesion volume was computed as area under the curve of lesion area *versus* distance from the aortic sinus^46^.

### Metabolomic analysis

#### Sample preparation

Ice-thawed plasma was extracted using an ice-cold mixture of 1:1 methanol:ethanol (v/v) containing 10 μM *L*-phenylalanine-d_8_ as the internal standard. The ratio of extraction solvent to plasma was 4:1 (v/v). The resulting mixture was vortexed for 2 min prior to centrifugation at 4 °C and 10,000 × g for 10 min. Pooled plasma extracts were run as a quality control measure to assess for system drift.

#### Chromatographic separation conditions

In order to expand the metabolomic coverage to both polar and non-polar metabolites in plasma, two 2.1 × 50 mm columns (SeQuant ZIC-cHILIC, 3 μm followed by Halo C8, 2.7 μm) were directly coupled in series as an orthogonal separation system under a single compatible solvent elution program, as previously described^41^. A 2 μL plasma extract was injected per run and analyzed with a linear gradient at a flow rate of 200 μL/min and a column temperature set at 40 °C. The LC system used was an Agilent 1200 RR series LC system (Agilent Technologies Inc., CA). Mobile phase A was 100% acetonitrile (LC-MS grade, Sigma Aldrich) and mobile phase B was 10 mM ammonium acetate (Sigma Aldrich) adjusted to pH 3 with formic acid. Gradient elution started with 95% A for 0.5 min and then linearly decreased to 30% A at 13 min, held at 30% A for 2 min followed by a ramping up to 95% A for 1 min. The columns were then re-equilibrated for 15 min at 95% A prior to subsequent sample analysis. Samples were analyzed in a random order.

#### Mass spectrometer parameters

Plasma extracts were analyzed by TOF-MS using a Bruker micrOTOF II (Bruker Daltonics) mass analyzer equipped with an electrospray ionization (ESI) source (Agilent Technologies Inc.). Each plasma extract sample was run separately in positive and negative electrospray modes. The TOF-MS system was operated with the following settings: capillary potential of +3800 V (ESI-) or −4500 V (ESI+), nebulizer gas (N_2_) pressure of 3.0 bar, dry gas (N_2_) flow rate of 6.0 L/min, a chamber temperature of 250 °C, mass range of 50–1000 m/z, and a scan rate of 2 Hz. A tuning mix solution was used for external mass calibration of the TOF-MS system daily.

#### Data analysis

The acquired mass spectra were calibrated internally using endogenous sodium formate clusters (Bruker Daltonics DataAnalysis 4.0). Calibrated LC-MS files were then converted to mzXML format (Bruker CompassXport) and processed with the XCMS and CAMERA software package (Scripps Institute for Metabolomics)^47^. This software provides retention time alignment, metabolite feature detection, feature matching, peak integration, adduct and isotope annotation. Metabolite feature is a chromatographic peak with a unique chromatographic retention time and a unique mass-to-charge (m/z) ratio. Peak areas integrated by XCMS were normalized with the internal standard *L*-phenylalanine-d_8_. Metabolite features which showed poor analytical reproducibility (CV > 30% in pooled samples) or eluted close to the solvent front that is prone to ion suppression (k’ < 0.4) were removed. To correct for age-associated features that altered in similar patterns in both mouse models; we removed metabolite features that were significantly altered (ANOVA *p*-value < 0.05) between the three time points in the ApoE^+/−^ control mice.

Metabolites were identified based on available authentic standards, tentatively assigned based on structural analogs or by matching their accurate mass/empirical formula with metabolite databases including METLIN, Human Metabolome Database and Lipid Map. In all cases, metabolites were annotated based on their characteristic m/z and retention time.

### Statistical analysis

Principal component analysis, partial least squares discriminant analysis, heat map, pathway analysis, one-way ANOVA and between-subject two-way ANOVA were performed using MetaboAnalyst 3.0^16^. Auto scaling followed by log transformation was applied in all multivariate analyses. Student t-test (two-tailed, unpaired heteroscedastic) and one-way ANOVA followed by Tukey’s HSD test were performed using Microsoft Excel 2010 and GraphPad Prism (v6.01), respectively.

## Additional Information

**How to cite this article**: Dang, V. T. *et al*. Comprehensive Plasma Metabolomic Analyses of Atherosclerotic Progression Reveal Alterations in Glycerophospholipid and Sphingolipid Metabolism in Apolipoprotein E-deficient Mice. *Sci. Rep.* **6**, 35037; doi: 10.1038/srep35037 (2016).

## Supplementary Material

Supplementary Information
