# Supplementary material for: Comprehensive Plasma Metabolomic Analyses of Atherosclerotic Progression Reveal Alterations in Glycerophospholipid and Sphingolipid Metabolism in Apolipoprotein E-deficient Mice
Source: Sci Rep. 2016 Oct 10;6:35037. doi: 10.1038/srep35037 (PMC5056345; doi:10.1038/srep35037)
Supplement: Supplementary Information [file srep35037-s1.pdf]

Comprehensive Plasma Metabolomic Analyses of Atherosclerotic Progression Reveal Alterations in Glycerophospholipid and Sphingolipid Metabolism in Apolipoprotein E-deficient Mice

Metabolomic Analyses of Atherosclerotic Progression

Vi T. Dang[1,2], Aric Huang[1,2], Lexy H. Zhong[1], Yuanyuan Shi[1], Geoff H. Werstuck[1,2,3]*

[1]Thrombosis and Atherosclerosis Research Institute, Hamilton, Ontario, Canada

[2]Department of Chemistry and Chemical Biology, McMaster University, Hamilton, Ontario, Canada

[3]Department of Medicine, McMaster University, Hamilton, Ontario, Canada

<u>**SUPPLEMENTARY FIGURES**</u>

Supplementary Figure S1: FPLC analysis of plasma lipoprotein profiles for ApoE$^{-/-}$ and ApoE$^{+/-}$ mice at 5, 10 and 15 weeks of age. Representative profiles derived from pooled plasma from 3 mice per group are shown.

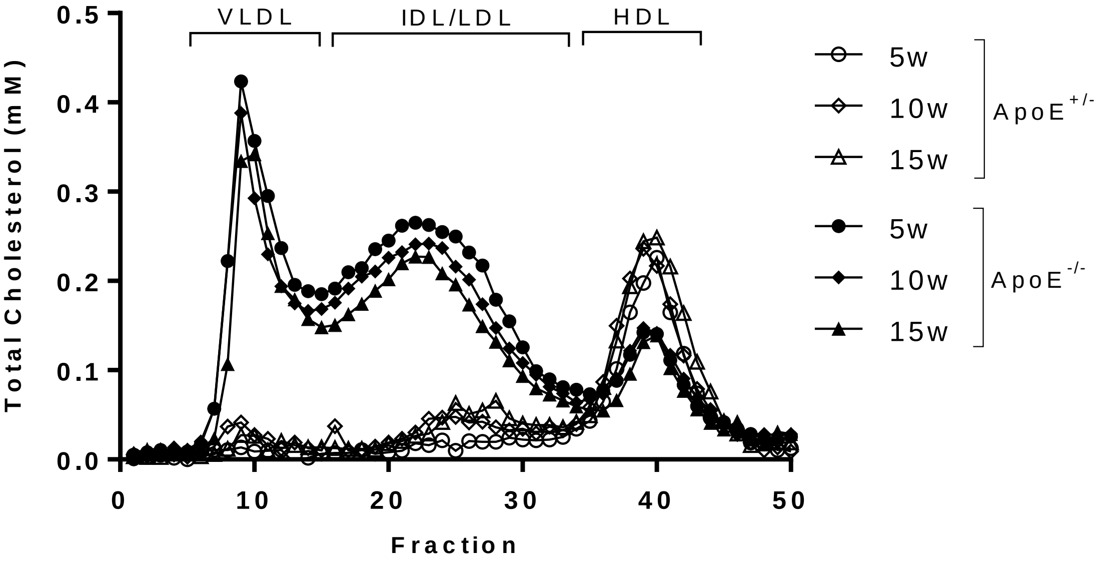

Supplementary Figure S2: ApoE$^{+/-}$ mice do not develop atherosclerosis at any age. Representative images of Masson's trichrome stained aortic cross-sections of ApoE$^{+/-}$ mice at 5, 10 and 15 weeks of age.

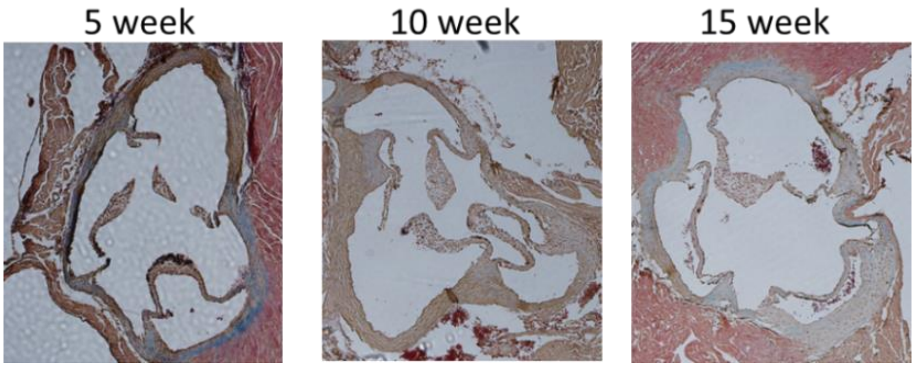

Supplementary Figure S3: Further characterization of atherosclerotic progression. (A) Representative images of aortic cross-sections of ApoE$^{-/-}$ mice at stage I and II of atherosclerosis, stained with Masson's trichrome or TUNEL. (B) Quantification of lesional collagen content and apoptotic cell death. Percentage of collagen was calculated by dividing positively stained areas by total lesion area. Number of apoptotic cells was measured in the cross-section of the maximal lesion area. Data are presented as the mean ±SD. n=5/group.

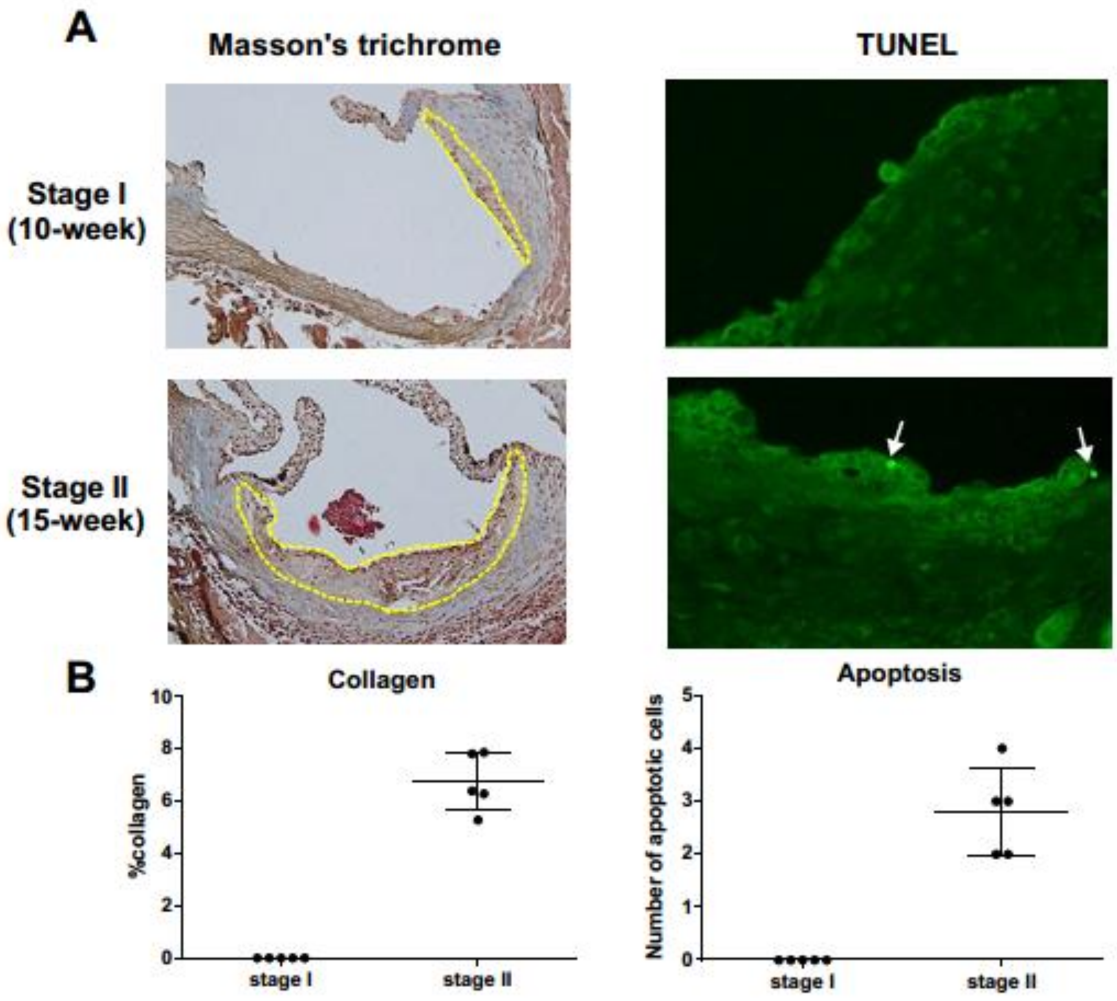

Supplementary Figure S4: Analytical quality assurance of instrumental performance. Tight clustering of pooled samples in the principle component analysis demonstrated good instrumental reproducibility throughout the period of analysis. D and H indicate ApoE$^{-/-}$ and ApoE$^{+/-}$, respectively. n=7 per ApoE$^{-/-}$ group, n=4-6 per ApoE$^{+/-}$ group and n=12 for pooled samples.

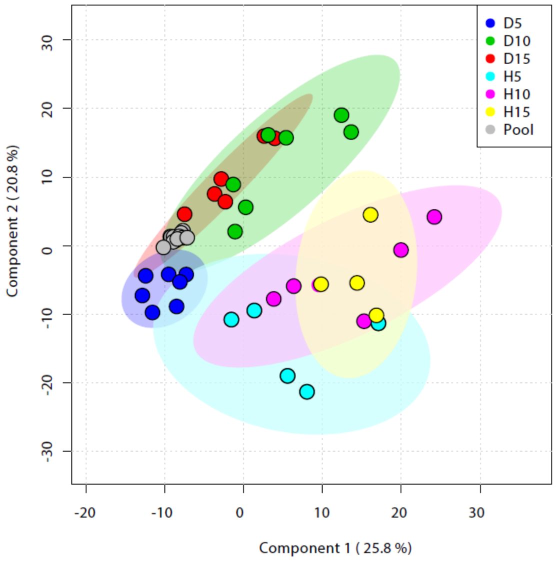

SUPPLEMENTARY TABLES

Supplementary Table S1: Analysis of metabolic parameters of ApoE$^{-/-}$ and ApoE$^{+/-}$ mice at 5, 10 and 15 weeks of age. **p*<0.05, n=7 per ApoE$^{-/-}$ group and n=4-6 per ApoE$^{+/-}$ group.

Model	Age (week)	Body weight (g)	Fasting glucose (mM)	Plasma triglycerides (mM)	Plasma cholesterol (mM)
ApoE$^{-/-}$	5	13.5±0.7	8.5±1.4	0.9±0.2	6.5±1.1
	10	18.7±1.2	7.7±1.0	1.0±0.2	6.3±1.3
	15	19.9±1.2	8.7±1.4	1.0±0.1	6.6±1.1
ApoE$^{+/-}$	5	13.4±0.6	7.9±0.8	0.8±0.2	2.6±0.4*
	10	17.1±1.5	8.9±1.5	0.8±0.1	2.4±0.9*
	15	20.2±1.9	8.8±1.2	0.7±0.2	2.2±0.5*

Supplementary Table S2: Identification of metabolites that were altered at a more extreme significance cut-offs (p-value <0.0001 and fold change >±5) in ApoE$^{-/-}$ mice, compared to the age-match ApoE$^{+/-}$ control mice. Fold change is relative to the age-matched control. RT: retention time, nd: not determined due to undetected levels in #ApoE$^{-/-}$ mice or *ApoE$^{+/-}$ mice.

m/z	RT (min)	Metabolite	5-week		10-week		15-week	
			p-value	fold change	p-value	fold change	p-value	fold change
(+) 728.525	10.0	PC(32:3)	7.4E-06	5.0	1.7E-06	5.7	1.3E-06	7.7
(+) 729.586	11.3	SM(d36:2)	7.1E-05	5.7	1.1E-09	9.2	3.3E-07	9.9
(-) 733.552	10.2	DAG(42:5)	5.5E-06	5.2	1.9E-08	5.8	1.6E-07	7.4
(+) 747.723	26.0	DAG(O-46:2)	7.4E-05	nd#	4.3E-07	nd#	9.4E-10	nd#
(+) 408.267	10.9	CAR(14:1-OH)	-	-	2.6E-11	7.6	4.2E-07	5.7
(+)442.260	10.9	PA(16:0)	-	-	3.8E-11	6.4	1.4E-07	5.7
(+) 725.554	10.8	SM(d34:1)	-	-	1.9E-09	5.6	5.4E-10	6.7
(+) 731.604	20.0	SM(d36:1)	-	-	3.9E-06	5.0	5.2E-06	6.3

(+) 826.662	5.6	PC(O-40:3)	-	-	5.2E-06	nd*	2.1E-07	nd*
(-) 893.699	11.0	PA(50:3)	-	-	1.0E-06	6.9	4.8E-06	9.3
(+) 797.662	6.5	TG(46:2)	1.0E-06	6.2	-	-	-	-
(+) 575.511	33.0	DAG(O-34:4)	-	-	1.5E-05	7.9	-	-
(-) 825.370	11.1	PIP(28:4)	-	-	2.0E-05	5.8	-	-
(+)839. 478	3.2	PG(40:9)	-	-	2.4E-05	5.8	-	-
(-) 883.541	10.9	PI(38:5)	-	-	1.1E-10	32.2	-	-
(+) 624.591	30.3	CAR(32:0)	-	-	-	-	3.7E-05	7.0
(+) 689.556	10.0	HexCer(d32:1)	-	-	-	-	4.6E-07	6.1
(+) 691.558	10.0	PA(O-36:0)	-	-	-	-	8.4E-07	6.6
(+) 700.570	3.6	HexCer(d34:1)	-	-	-	-	2.8E-06	6.7
(+) 711.539	10.0	PA(O-36:1)	-	-	-	-	4.6E-06	5.9
(+) 727.523	10.3	PA(36:0)	-	-	-	-	1.2E-05	5.2
(+) 729.523	10.0	PE(34:4)	-	-	-	-	5.0E-06	5.0
(-) 735.528	9.7	PA(O-40:5)	-	-	-	-	3.2E-06	6.5
(+) 739.529	4.0	DAG(44:10)	-	-	-	-	1.9E-06	5.5
(+) 741.529	10.6	DAG(44:9)	-	-	-	-	2.7E-05	6.8
(+) 742.535	10.6	PE(36:3)	-	-	-	-	1.0E-05	78.5
(+) 772.533	5.3	PE(O-38:6)	-	-	-	-	1.0E-08	6.0
(+) 793.546	11.0	Hex_2Cer (d28:2)	-	-	-	-	2.1E-08	5.0

CAR: acylcaritine, Cer: ceramide, DAG: diacylglycerol, HexCer: hexosyl-ceramide, PA: phosphatidic acid, PC: phosphatidylcholine, PE: phosphatidylethanolamine, PG: phosphatidylglycerol, PI: phosphatidylinositol, PIP: PI-phosphate, SM: sphingomyelin, TG: triacylglycerol.

Supplementary Table S3: A total of 137 metabolites were found to be significantly altered (ANOVA $p<0.01$ followed by Fisher's LSD *post hoc* test) between the three stages of atherosclerosis in ApoE$^{-/-}$ mice. These metabolites were identified based on available authentic standards (level 1 identification), tentatively assigned based on structural analogs (level 2 identification), or by matching their accurate mass/empirical formula within metabolite databases including METLIN, Human Metabolome Database and Lipid Map (level 3 identification). RT: retention time, LSD: Least Significant Difference.

m/z	RT (min)	p-value	Fisher's LSD	Metabolite	Identification Level
(+)90.092	8.9	9.40E-03	5w-10w; 5w-15w	Dimethylethanolamine	2
(+)110.071	14.4	5.30E-04	5w-10w; 5w-15w	Glycerol	2
(+)112.053	8.4	4.30E-05	5w-10w; 5w-15w	Cytosine	1
(+)116.071	14.9	7.40E-03	5w-10w; 5w-15w	Acetamidopropanal	2
(-)119.041	10.6	6.80E-03	10w-5w; 15w-5w	Deoxythreonic acid	2
(+)130.050	11.9	4.20E-04	5w-10w; 5w-15w	N-Acryloylglycine	2
(+)130.084	8.9	6.50E-03	5w-10w; 5w-15w	Pipecolic acid	2
(+)130.087	14.6	2.10E-04	5w-10w; 5w-15w	Acetylaminobutanal	2
(+)131.082	14	8.90E-03	5w-10w	Pyrroline-carboxylic acid	2
(+)132.066	14	5.70E-03	5w-10w; 5w-15w	Propionylglycine	2
(+)132.102	4.6	1.10E-03	5w-10w; 5w-15w	FA(6:1)	1
(+)140.068	8.5	7.10E-06	5w-10w; 5w-15w	Dihydroxybenzylamine	2
(-)145.064	11.9	1.70E-04	5w-10w; 5w-15w	Glutamine	1
(+)147.113	14.6	2.50E-04	5w-10w; 5w-15w	Lysine	1
(+)148.096	8.8	1.40E-03	5w-10w; 5w-15w	Isoleucine	1

(+)156.042	8.5	8.50E-05	5w-10w; 5w-15w	N-Methylethanolaminium phosphate	2
(+)156.077	14.4	1.30E-03	5w-10w; 5w-15w	Histidine	1
(-)159.067	2.9	9.80E-03	5w-10w; 5w-15w	Methyladipic acid	2
(-)159.115	14	5.30E-03	5w-10w; 5w-15w	Methyl-Lysine	1
(+)161.085	14	4.30E-04	5w-10w; 5w-15w	Alanyl-Alanine	2
(-)164.073	9	2.30E-03	5w-10w; 5w-15w	Phenylalanine	1
(+)169.059	11.9	7.70E-05	5w-10w; 5w-15w	Pyrroloyl-Glycine	2
(-)172.992	2.2	1.50E-04	10w-5w; 15w-5w	Phenol sulphate	2
(+)175.119	14.6	2.00E-04	5w-10w; 5w-15w	Arginine	1
(-)181.072	9.8	2.60E-03	5w-10w; 5w-15w	Hexitol (C6H14O6)	2
(+)185.033	11.9	4.60E-05	5w-10w; 5w-15w	Hydroxymethylglutaric acid	2
(+)187.073	14	9.20E-04	5w-10w; 5w-15w	Pyroglutamylglycine	2
(-)203.103	14	8.70E-03	5w-10w; 5w-15w	Serinyl-Valine	2
(+)206.049	2.5	7.90E-03	10w-15w; 10w-w	p-Cresol sulfate	3
(+)208.051	12.9	3.20E-04	5w-10w; 5w-15w	Oxalosuccinate	2
(+)217.083	12.5	1.00E-02	5w-10w; 5w-15w	Hydroxyphenyl-valeric acid	2
(+)229.154	9.4	3.60E-03	10w-5w; 15w-5w	Isoleucyl-Proline	2
(-)235.146	4	3.90E-03	10w-5w; 10w-15w	FA (12:0)	1
(+)241.130	14.4	2.40E-03	10w-5w; 15w-5w	Alanyl-methyl-Histidine	2
(+)247.092	14	7.20E-03	5w-10w; 5w-15w	Aspartyl-Hydroxyproline	2
(-)257.004	3.9	9.20E-03	10w-15w; 5w-15w	Phosphonoglucono-lactone	2
(+)267.136	11.5	3.20E-03	5w-10w; 5w-15w	Threoninyl-Phenylalanine	2
(+)273.121	8.4	1.30E-03	5w-10w; 5w-15w	5C-Aglycone	2

(+)275.118	8.4	3.00E-03	5w-10w; 5w-15w	Glutamyl-Glutamine	2
(-)282.155	5	3.70E-03	5w-10w; 5w-15w	Lysyl-Histidine	2
(-)309.169	3.3	1.80E-03	5w-10w; 5w-15w	FA (18:5)	2
(+)315.079	14	8.50E-03	5w-10w	Cysteinylglycine disulfide	2
(-)336.099	4.3	2.90E-03	5w-10w; 5w-15w	S-Hydroxymethylglutathione	2
(+)361.170	8.5	7.80E-03	5w-10w; 5w-15w	Leukotriene derivative	3
(+)370.054	14.4	5.50E-03	10w-5w	Adenosine monophosphate	1
(+)377.144	5.1	6.50E-04	5w-10w; 5w-15w	Riboflavin	2
(+)380.254	9.9	2.90E-03	5w-10w; 5w-15w	Sphingosine 1-phosphate	3
(+)390.958	10.9	3.50E-03	5w-10w; 5w-15w	Phosphoribosyl pyrophosphate	2
(+)391.283	2.4	1.80E-03	15w-10w; 5w-10w	Ketolithocholic acid	2
(-)398.124	4.3	3.50E-03	5w-10w; 5w-15w	S-Adenosylmethionine	2
(+)404.313	2.2	2.50E-03	5w-10w; 5w-15w	Glycerol trihexanoate	1
(+)423.299	3.5	3.90E-03	15w-10w; 5w-10w	DHAP(18:0e)	3
(+)428.372	6.1	9.90E-03	5w-10w; 5w-15w	CAR(18:0)	2
(+)429.294	4.8	6.40E-03	5w-10w; 5w-15w	MAG(22:4)	2
(-)435.240	10.2	1.60E-04	15w-10w; 5w-10w; 15w-5w	Lyso-PA(18:1)	2
(-)457.234	8.9	3.80E-03	5w-10w; 5w-15w	Sulfodeoxycholic acid	2
(-)463.304	2.2	1.60E-04	5w-10w; 5w-15w	FA (28:6-OH)	2
(-)464.314	8.8	1.70E-03	15w-5w; 15w-10w	Lyso-PE(P-18:0)	2
(+)470.416	6.4	8.40E-03	5w-15w; 10w-15w	Cer(t28:1)	3
(-)477.308	2.2	3.60E-04	5w-10w; 5w-15w	Steroid derivative (C29H46O3)	3
(+)532.294	8.1	2.30E-03	5w-10w; 5w-15w	Lyso-PS(P-18:0)	2
(+)539.337	3.6	8.90E-03	5w-10w; 15w-	Lyso-PG(20:1)	2

			10w		
(+)540.366	8.7	3.70E-03	15w-5w; 15w-10w	Lyso-PS(O-20:0)	2
(-)552.498	9.3	4.90E-03	5w-10w; 5w-15w	Cer(t34:1)	3
(-)557.458	2.9	8.90E-03	5w-10w; 5w-15w	FA (34:1-OH)	2
(-)558.333	8.3	6.90E-03	10w-5w; 15w-5w	CerP(t26:0)	3
(-)568.362	8.4	4.00E-03	10w-5w; 15w-5w	CerP(d28:1)	3
(+)574.480	2.5	9.40E-03	5w-10w	Cer(t34:2)	3
(+)578.512	3.5	7.90E-03	5w-10w; 5w-15w	Cer(t34:0)	3
(+)580.903	10.9	5.00E-03	5w-10w	Inositol pentaphosphate	2
(-)585.353	8.4	2.20E-03	10w-5w; 15w-5w	PA(28:3)	2
(+)586.520	4.3	7.10E-05	15w-5w; 15w-10w	Cer(d36:2)	3
(+)596.369	9.1	3.50E-05	5w-10w; 5w-15w	PS(22:0)	2
(+)606.447	9	4.20E-03	5w-10w	Lyso-PC(24:1)	2
(+)608.463	9.8	4.40E-03	5w-10w; 15w-10w	Lyso-PC(24:0)	2
(+)613.417	8.3	3.70E-04	5w-10w; 5w-15w	Ubiquinone-6	3
(+)624.591	30.3	4.10E-03	10w-5w; 15w-5w	CAR(32:0)	2
(-)630.425	9.4	8.40E-04	5w-10w; 5w-15w	PE(28:2)	2
(-)632.417	9.4	5.10E-03	5w-10w; 5w-15w	PE(28:1)	2
(+)637.419	8.3	1.10E-03	5w-10w; 5w-15w	PG(26:1)	2
(+)638.406	8.3	7.60E-04	5w-10w; 5w-15w	Lyso-PE(30:6)	2
(+)639.426	8.3	1.60E-04	5w-10w; 15w-10w	PG(26:0)	2
(-)640.278	2.2	7.00E-04	10w-5w; 15w-5w	Leukotriene	3
(-)641.525	30.5	3.80E-04	10w-5w; 15w-5w	DAG(38:5)	2
(-)643.398	9	1.70E-03	5w-10w; 5w-15w	Lyso-PG(28:4)	2
(-)657.378	9	1.80E-03	5w-10w; 5w-	PG(28:4)	2

			15w		
(-)658.586	27.1	2.70E-03	10w-5w; 10w-15w	Cer(d40:0)	3
(-)659.376	9.1	1.10E-03	5w-10w; 5w-15w	Lyso-PA(32:5)	2
(+)673.566	2.8	9.90E-03	5w-10w; 5w-15w	DAG(38:1)	2
(-)677.268	2.3	1.00E-03	10w-5w; 15w-5w	Lyso-PIP(18:1)	2
(+)679.631	3.4	4.40E-03	5w-10w; 5w-15w	DAG(40:1)	2
(+)689.556	10	1.90E-04	15w-5w; 15w-10w	HexCer(d32:1)	3
(+)691.558	10	2.80E-03	15w-5w; 15w-10w	PA(O-36:0)	2
(+)697.428	3.3	5.50E-03	10w-5w; 15w-5w	Lyso-PG(30:3)	2
(-)698.558	4	2.40E-03	5w-10w; 15w-10w	CerP(d40:2)	3
(-)706.355	9.4	5.70E-04	5w-10w; 5w-15w	PS(28:4)	2
(+)706.667	2.5	4.90E-03	5w-10w; 5w-15w	DAG(O-42:3)	2
(+)707.547	10.3	8.30E-04	5w-10w; 5w-15w	PE(32:1)	2
(-)708.354	9.4	5.70E-04	5w-10w; 5w-15w	PS(28:3)	2
(+)710.024	2.3	5.80E-03	10w-5w; 15w-5w	Uridine Diphosphate Acetylhexosamine Sulfate	2
(+)711.539	10	7.20E-04	15w-5w; 15w-10w	PE-Cer(d36:1)	3
(+)717.546	9.7	4.60E-05	5w-10w; 5w-15w	SM(d34:1-OH)	2
(+)717.573	4.5	4.20E-03	5w-10w; 15w-10w	PA(O-38:1)	2
(+)719.567	11.2	2.60E-04	5w-10w; 5w-15w	Cholesteryl Ester (22:6)	2
(-)720.523	3.8	3.20E-03	15w-5w; 15w-10w	Lyso-PS(32:0)	2
(+)727.523	10.3	5.10E-03	15w-5w; 15w-10w	PA(36:0)	1
(+)728.557	21.9	8.30E-04	10w-5w; 15w-5w; 15w-10w	PE(O-36:3)	2
(-)728.560	22.9	2.20E-03	10w-5w; 15w-5w	PE(O-36:2)	2

(+)730.535	9	5.00E-04	5w-10w; 5w-15w; 15w-10w	PC(32:2)	2
(+)731.540	9	6.80E-04	5w-10w; 5w-15w; 15w-10w	PE(34:3)	2
(+)732.546	9	6.80E-03	5w-10w	PC(32:1)	2
(-)733.552	10.2	8.90E-06	15w-10w; 15w-5w	DAG(42:5)	2
(+)734.567	21.5	1.50E-03	5w-10w; 5w-15w	PC(32:0)	1
(-)736.527	3.4	1.90E-04	5w-10w; 5w-15w	CerP(d40:1)	3
(+)739.512	9.6	1.90E-03	5w-10w; 5w-15w	PC(32:6)	2
(+)740.533	3.2	9.20E-03	5w-10w; 15w-10w	PG(32:0)	1
(+)744.546	9.9	2.10E-06	5w-10w; 5w-15w	PE(36:2)	1
(+)755.554	3.4	8.00E-05	5w-10w; 5w-15w	PA(40:3)	2
(-)768.541	5.3	4.10E-03	10w-5w; 15w-5w	PE(38:3)	2
(+)772.533	5.3	8.60E-03	15w-5w; 15w-10w	PE(O-38:6)	2
(+)776.492	3.3	3.80E-04	5w-10w; 5w-15w	SHexCer(d34:3)	3
(+)794.511	9.5	3.90E-05	5w-10w; 5w-15w	SHexCer(t34:2)	3
(+)794.601	20.1	8.80E-03	5w-10w	PC(O-36:2)	2
(+)796.523	11.9	2.20E-03	5w-10w; 5w-15w	PE(40:7)	2
(+)797.662	6.5	4.60E-03	15w-10w; 5w-10w	TAG(46:2)	2
(-)804.576	21.7	4.30E-03	5w-10w; 5w-15w	HexCer(t38:2)	3
(-)806.582	21.8	7.50E-03	5w-10w; 5w-15w	CerP(t44:2)	3
(+)808.663	5.9	6.50E-04	5w-10w; 15w-10w	HexCer(d42:3)	3
(+)809.644	21.4	6.50E-04	5w-10w; 5w-15w	SM(d40:1)	2
(+)818.603	20.6	9.00E-04	15w-5w; 15w-10w	PC(O-38:4)	2
(-)820.492	3.3	2.30E-03	5w-10w; 5w-15w	PS(36:3)	2
(+)820.617	21.7	2.00E-03	15w-5w; 15w-	PS(38:0)	2

			10w		
(+)821.561	8.9	9.40E-04	15w-5w; 15w-10w	PI(O-34:2)	2
(+)821.619	22	3.10E-03	15w-5w; 15w-10w	PE(O-42:7)	2
(+)848.634	6.7	9.60E-03	15w-5w; 15w-10w	PS(40:0)	2
(+)871.609	11.9	3.10E-04	5w-10w; 5w-15w	PC(42:10)	2
(-)902.627	11.9	7.90E-03	5w-10w; 5w-15wpa	PS(P-46:6)	2

CAR: acyl carnitine, Cer: ceramide, CerP: ceramide phosphate, DAG: diacylglycerol, FA: fatty acid, HexCer: hexosyl ceramide, MAG: monoacylglycerol, PA: phosphatidic acid, PC: phosphatidylcholine, PE: phosphatidylethanolamine, PG: phosphatidylglycerol, PI: phosphatidylinositol, PS: phosphatidylserine, SHexCer: sulfatide, SM: sphingomyelin, TAG: triacylglycerol.

Supplementary Table S4: Pathway analysis identified 29 affected pathways that were potentially affected during the progression of atherosclerosis.

Pathway Name	*p*-value	Impact value
Glycerophospholipid metabolism	0.01	0.30
Sphingolipid metabolism	0.02	0.31
Arginine and proline metabolism	0.04	0.09
Pentose phosphate pathway	0.10	0.02
Phenylalanine, tyrosine and tryptophan biosynthesis	0.11	0.50
Steroid biosynthesis	0.27	0.07
Phenylalanine metabolism	0.28	0.41
Purine metabolism	0.31	0.07
Histidine metabolism	0.36	0.24
Glycerolipid metabolism	0.41	0.28
Alanine, aspartate and glutamate metabolism	0.51	0.15
Cysteine and methionine metabolism	0.55	0.07
Inositol phosphate metabolism	0.56	0.06
Steroid hormone biosynthesis	0.89	0.06
Nitrogen metabolism	0.03	0
Lysine biosynthesis	0.11	0
D-Glutamine and D-glutamate metabolism	0.14	0
Biotin metabolism	0.14	0
Linoleic acid metabolism	0.16	0
alpha-Linolenic acid metabolism	0.23	0
Methane metabolism	0.23	0
Riboflavin metabolism	0.28	0
Pyrimidine metabolism	0.33	0
beta-Alanine metabolism	0.39	0
Citrate cycle (TCA cycle)	0.45	0
Lysine degradation	0.49	0
Galactose metabolism	0.54	0
Arachidonic acid metabolism	0.66	0
Fatty acid biosynthesis	0.72	0
